# Powder Pre-Treatment for Aerosol Deposition of Tin Dioxide Coatings for Gas Sensors

**DOI:** 10.3390/ma11081342

**Published:** 2018-08-02

**Authors:** Dominik Hanft, Murat Bektas, Ralf Moos

**Affiliations:** Department of Functional Materials, University of Bayreuth, 95447 Bayreuth, Germany

**Keywords:** Aerosol Deposition method (AD, AD method, ADM), SnO_2_ gas sensor, room temperature impact consolidation, crystallite size, powder treatment

## Abstract

The Aerosol Deposition (AD) method has the unique property to allow for manufacturing dense ceramic films at room temperature. As found in many publications, the deposition process is very sensitive to powder properties. In particular, powders of nano-sized particles and grains, e.g., from precipitation, are usually beyond the conventional size range of AD processability, yielding chalk-like films of low mechanical stability. Thus, the conventional AD process is limited in applicability. In this study, we try to overcome this problem by adapting the standard milling treatment of powders for improved deposition by additional temperature pre-treatment. Using commercial tin dioxide and including a temperature treatment for grain growth, makes the powder accessible to deposition. In this way, we achieve optically translucent and conductive SnO_2_ thick films. With the application such as a gas sensitive film as one of many possible applications for SnO_2_ thick-films, the sensors show excellent response to various reducing gases. This study shows one exemplary way of extending the range of adequate powder and applications for the AD method.

## 1. Introduction

A coating technique that was developed recently is the aerosol deposition (AD) method using a room temperature impact consolidation process (RTIC) [1,2]. It allows for processing of ceramic films at room temperature directly from a dry ceramic powder. No firing of the deposited film is required as in the case of classical thick-film techniques, thus prohibiting material reactions [3]. Expensive vacuum equipment is not necessary. In the AD process, the powder is transferred into an aerosol by means of a carrier gas and transported to a vacuumed deposition chamber (some mbar) by a pressure difference. Accelerated to several hundred m/s in a nozzle, the aerosol jet impacts on a substrate material, forming dense coatings [1,3,4]. Several articles have been published about parameters that affect the film formation and morphology. Besides process parameters like carrier gas composition [5], chamber pressure, nozzle geometry and particle velocity [6,7], the main influential film formation parameter is the powder itself. In most cases, it is characterized by the particle size and distribution of starting powder particles [1,4,8,9,10]. There are several publications about powder pre-treatment to improve deposition onto dense and mechanically stable films [11,12,13]. However, AD seems to work well only for particle sizes of approximately 200 nm up to 2 µm. When only nano-sized powders are available, as for some materials, e.g., synthesized by precipitation route, resulting films are porous and chalk-like with insufficient mechanical stability [1], which means AD is not applicable for this material.

Concerning the application point of view, one would sometimes prefer thin films of nano-sized particles and crystallites, e.g., for improved sensitivity of gas sensors or as a µm-thick transparent conductive layers. Here, we try to achieve this by AD. There have been reports on the deposition of gas sensing materials [3,14,15,16,17,18,19]. To our knowledge, up to now no report on the deposition of n-type semiconducting SnO_2_ with the AD method has been published. Metal oxide semiconducting SnO_2_ is used in a variety of applications. Besides the transparency, the low resistance in the doped state, and its catalytic activity, it is the main material for conductometric gas sensors [20,21]. Much research was conducted to understand and improve the sensing behavior of SnO_2_-based sensors [21,22,23]. Research includes doping strategies [24,25,26], morphology variations, appropriate sensor designs [26,27,28] with the intention to improve sensitivity, selectivity, and stability [27,28,29,30]. High sensitivity, low cost, fast response and easy miniaturization are the main advantages of semiconducting SnO_2_ gas sensors. State-of-the-art techniques to manufacture gas sensitive layers are sputtering [31], spin coating [32], or screen printing [33]. Extensive summaries can be found in [22,23,26]. However, it has to be ensured that the sensitive layer is of sufficient mechanical stability. In conventional methods, this is done by post-sintering of, e.g., screen-printed films.

In this study, we modified the powder properties by a thermal process and a subsequent milling pre-treatment to improve the deposition behavior. As a result, we could achieve stable films of tin (IV) oxide. The gas sensing properties of these films were investigated with the aim of a basic suitability as a gas sensitive film. With this, we show the AD of tin dioxide and its applicability to manufacture gas sensor films.

## 2. Materials and Methods

For deposition tests, we used a commercially available SnO_2_ powder from Alfa Aesar (99.9%). The deposition behavior was investigated for different additional preparation steps of the powder: (a) Solely drying of the powder at 200 °C, (b) drying and additional sintering of the powder at 1250 °C in air and (c) drying, sintering and subsequent planetary ball milling in zirconia jars with cyclohexane and zirconia balls for 4 h. For good comparability, all powders were sieved (90 µm mesh size) and ultimately dried at 200 °C for at least 24 h prior to deposition.

**Figure 1:**
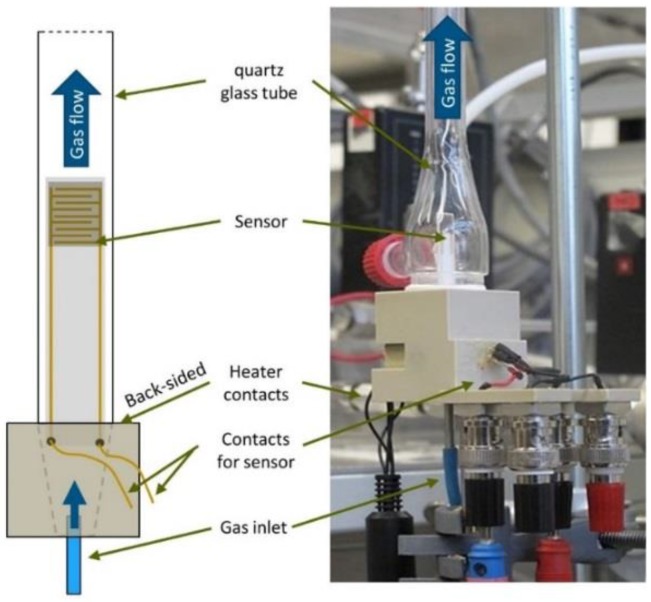
Gas sensor test rig and setup as schematic (**left**) and image (**right**): Sample in sample holder with sealed glass tube, gas inlet, outlet and electrical contacts for heating and electrical measurement.

The AD device used for the deposition tests is described in [4]. The films were deposited on alumina substrates with screen-printed Au-interdigital electrodes (IDEs). Coated samples for gas sensing tests were annealed at 600 °C for 5 h prior to testing to obtain stable sensing signals at elevated operation temperatures. During the gas-sensing experiments, the coated structure was heated directly by means of a backside heater structure (also screen-printed). In order to determine the gas-sensing properties of the SnO_2_-coated sensor transducer, they were contacted in a sample holder (see Figure 1) with six contact pins. The platinum electrodes of the heater were connected to a heater controller with four contact pins and the two gold electrodes on the front side of substrate were connected to a Keithley 2700 multimeter. The sample holder was mounted in a small quartz-glass tube.

For further investigations concerning phase purity and crystallinity of powders and films, X-ray diffraction analysis (XRD) was conducted using CuK*α* radiation. Powder and film morphology was investigated by Scanning Electron Microscopy (SEM analysis). Particle size distribution for powders was investigated by Malvern Mastersizer 2000. Powders were dispersed in H_2_O with a squirt of surfactant under ultrasonic treatment for de-agglomeration.

## 3. Results and Discussion

### 3.1. Powder Preparation

**Figure 2:**
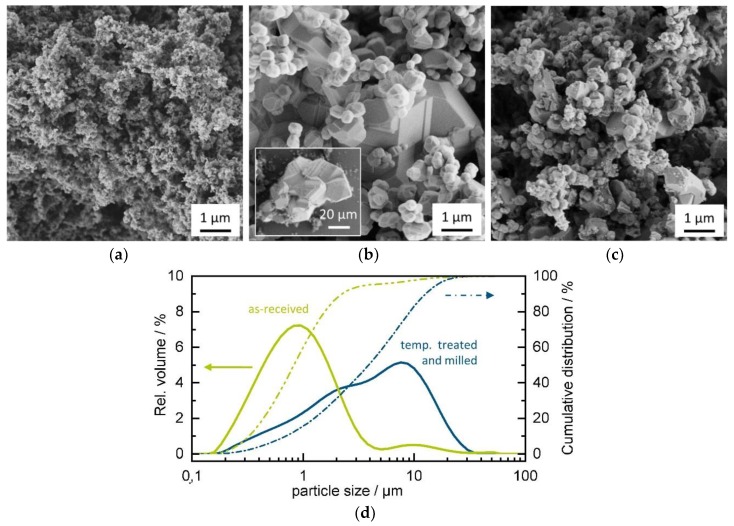
(**a**) SnO_2_ powder as-received, (**b**) powder after temperature treatment at 1250 °C in air, (**c**) temperature-treated powder after ball milling, and (**d**) particle size distribution for as-received and fully treated powders (solid lines show the relative volume, dotted lines the cumulative distribution).

Three powders of the same initial powder lot but differently prepared were used for aerosol deposition. SEM pictures of these powders are shown in Figure 2. The untreated bulk powder shows particles of size below 100 nm (Figure 2a). Powders with such small particle size usually show poor deposition behavior resulting in chalk-like films of loosely compacted powders and without mechanical stability [1]. To increase the particle size of the initial powder, a temperature of 1250 °C was chosen because it was found that SnO_2_ tends to decompose at above 1300 °C [34]. The particle size increased strongly (to a range of around 200 nm to several µm) whereby the size distribution became broader as well, since particles of several tens of microns in size can be found (Figure 2b). Through the temperature treatment, large single particles >20 µm are formed as well as large aggregates of particles connected by sinter necks. By ball milling of this powder, bigger particles and the large aggregates are fractured into smaller crystals as can be seen from Figure 2c. Prior to the here-shown treatment, we conducted a study (not shown here) to find the minimum treatment temperature to obtain particles of adequate size for deposition. Sinter temperatures up to 1000 °C were found to yield still particles below 200 nm but already of severe clustering by sinter necks, which supposedly will cause unsatisfying deposition results [9]. With this, a treatment at significantly lower temperatures compared to the one used in this study seems not be reasonable.

Particle size distribution for the as-received and the fully treated powder are given in Figure 2d. The results show a clear increase of the particle size through the temperature treatment. The *D*_50_ increases from 0.83 µm (as-received) to 3.9 µm for the fully treated powder. However, the particle size data for the as-received powder have to be handled with care, since the lower size limit (resolution) for the particle size measurement is in the range of the main particle size of the as-received powder (Figure 2a). One has to consider that the ultrasonic treatment prior to the measurement is inefficient in powder de-agglomeration since there are still significant amounts of powder in the 10-µm range for the size measurement, due to the extreme agglomeration behavior of nano-sized particles. However, both methods together give a good impression of the powders used in the following experiments.

### 3.2. Deposition of Tin Dioxide Films

Before deposition tests, all powders were dried at 200 °C for at least 24 h. Deposition experiments were conducted with parameters listed in Table 1. Parameters are given in ranges since they were varied in different experiments.

**Figure 3:**
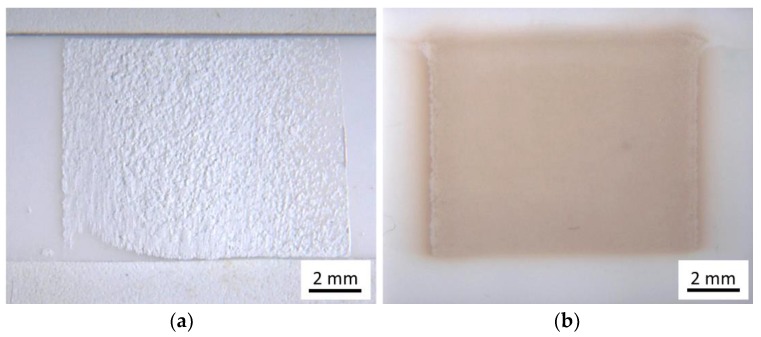
(**a**) Poor adhering and chalk-like AD film produced with untreated powder and (**b**) well adhering dense film deposited with temperature-treated and ball-milled powder before heat treatment.

Deposition tests were conducted on plane alumina substrates with and without interdigital electrode structures. Neither with the as-received (just dried) powder, nor with the temperature-treated powder without ball-milling film formation was possible. While we found films of loosely compacted powder for the case of untreated powder (Figure 3a), there was no film detectable by a stylus instrument in the case of the sintered powder but just a change of the treated substrate spot to a brownish color. In contrast to that, good deposition and homogeneous films could be achieved using the fully treated powder (see Figure 3b). The brown color of the film in the as-deposited state disappeared when annealed at 600 °C.

The chalk-like films are of poor mechanical stability and can be wiped off easily. In order to prevent continuous degradation and sensor signal drift due to, e.g., loose contacts, one has to guarantee good adhesion to both electrodes and substrate. Furthermore, a certain particle connection is required for sensing effects, which is hardly given for the film of loose powder compact in the case of the as-received powder.

**Figure 4:**
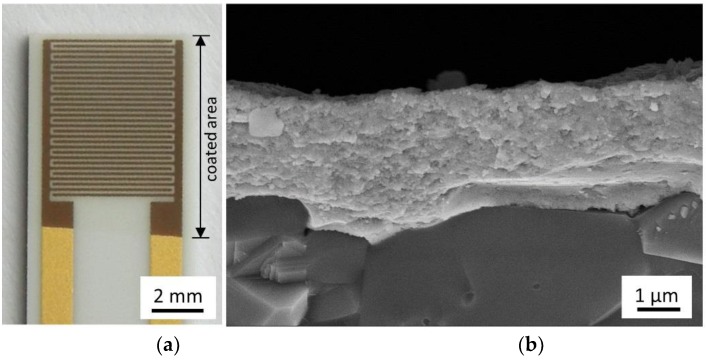
(**a**) Coated and at 600 °C annealed IDE structure and (**b**) SEM micrograph of fractured cross section of film on Al_2_O_3_ substrate.

Figure 4a shows an image of a coated and annealed IDE structure. The coated Au structure has a brown color, which is due to changed light scattering through the coating. On the alumina, the annealed film appears transparent.

In fact, Figure 4b shows a homogeneous coating on the substrate. The coating has low to no porosity and a nano-crystalline morphology that is typical for films of a well-working aerosol deposition process [1]. In this case, the thickness of the coating is around 2–3 µm. This suggests a deposition process as it is thought to result from deformation and fracture of particles into nanoscale crystallites [1].

The high impact of powder pre-treatment on the deposition quality has already been observed before. Akedo showed that powders with too small primary particles (as is the case for the as-received SnO_2_ powder) result in chalk-like and highly porous films of low mechanical stability [1]. By an additional sintering step, we increased the particle and crystallite size. However, deposition also did not work for the solely sintered powder. That may be because there are still large particles and sintered aggregates in the powder (see Figure 2b). Such particles of several tens of microns in diameter are thought to hinder film formation by damaging it in a way comparable to sand blasting [8,9]. By milling the powder and crushing larger particles, film formation and growth became possible. The positive effect of milling treatment on the deposition rate has been shown already in the literature [12,13]. It is thought to originate from pre-cracking of the particles and the formation of lattice defects that might support film formation through the reduction of the fracture energy. In the here-shown case, milling has the additional effect of eliminating clusters of sintered particles as well as large particles of high kinetic energy that might harm film formation [8,9]. The additional milling step seems inevitable to separate particles. A more extensive study on different annealing and milling parameters would be necessary to answer the question whether appropriate deposition would be possible without subsequent milling.

**Figure 5:**
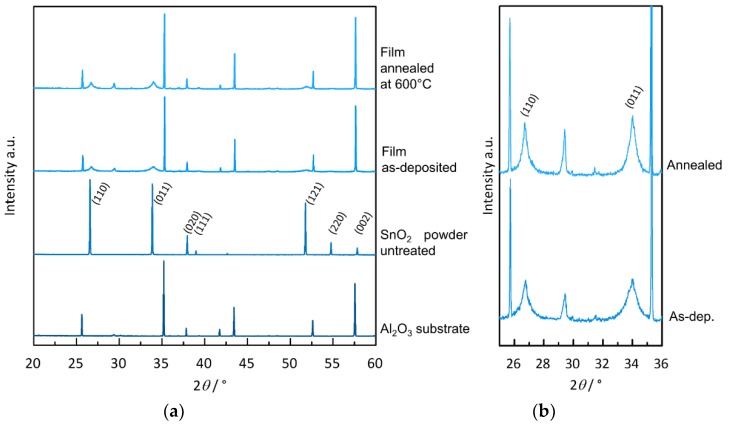
(**a**) XRD spectra of Al_2_O_3_ substrate, untreated SnO_2_ powder, as-deposited film as well as film annealed at 600 °C with equalized maximum intensities and (**b**) detailed view of the film patterns; (Miller indices are given for main SnO_2_-reflexes).

In order to check that the powder composition has not changed during the whole processing from thermal treatment at 1250 °C, over subsequent milling to deposition, XRD measurements have been conducted (see Figure 5a,b). XRD spectra are shown for alumina substrate material, powder, and films of SnO_2_, one as-deposited and one annealed at 600 °C. A certain reflex shift can be found, which could be attributed to a height mismatch in the measurement of films and powders. The significant reflexes in the given 2*θ*-range for SnO_2_ can also be observed for the AD film, although the reflexes are strongly broadened. The effect of reflex broadening is typical for AD films resulting from the decrease of crystal size and the appearance of microstrain through the strong mechanical imposition of particles during impact and film formation [1,2]. A more detailed view of the film patterns in Figure 5b depicts the broadened reflexes. The reflex at ~29.5° is not related to the film but might have its origin in the alumina substrate. Through temperature treatment at 600 °C in air, the SnO_2_ reflexes become slightly sharper. The additional sharp peaks in the film patterns can be assigned to the alumina substrate on which the film was deposited.

The mean crystallite size *τ* in the films and powders was calculated from the width of the 100% reflex using Scherrer’s Equation (1) [35]. The parameter *K* is a shape factor (approx. 0.9), λ is the wavelength of the X-ray, *β* is the reflex width at half-maximum intensity (FWHM) and *θ* is the Bragg angle.

(1) $$\tau =\frac{K\cdot \lambda }{\beta \cdot \cos\left(\theta \right)}$$

The calculated crystallite sizes of SnO_2_ for initial powder (untreated), 1250 °C heat-treated powder, untreated AD coating and heat-treated AD coating at 600 °C are about 61 nm, 126 nm, 17 nm, and 22 nm, respectively. The heat treatment of the initial powder doubles the mean crystallite size. One has to mention that the calculated crystallite size for the annealed powder seems to divert from the particle size obtained from the SEM image in Figure 2b, indicating that the particles are polycrystalline. With <30 nm, the crystallites in the film are typical for AD films, indicating a well-working film formation by the impact consolidation. The crystallite size ratio between deposited powder and film gives a value of approximately 7.5.

As we found in our small study, the particle and crystallite sizes seem to have a tremendous impact on the deposition behavior and film morphology. A much more detailed quantitative study gives a fundamental picture of the influence of the powder pre-treatment [36]. It gives a theoretical approach of the required crystallite and particle size for a successful film formation by the event of room-temperature impact consolidation (RTIC) through particle fracture. The reduction of the crystallite size through fracture and the corresponding ratio of crystallite size in powder and film can serve as an indicator for successful film formation and consolidation. The here-given value of 7.5 approaches the necessary range for deposition by RTIC.

### 3.3. Gas Sensing Properties of AD Coated SnO_2_ Sensors

**Figure 6:**
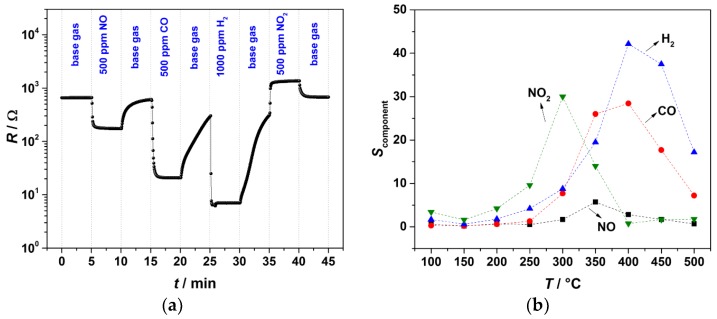
(**a**) Resistance change of AD-coated SnO_2_ sensor at 400 °C in the presence of different gas components in flowing base gas (20% O_2_ in N_2_ and 3% H_2_O) and (**b**) response of the AD-coated SnO_2_ sensor towards the gas components between 100 °C and 500 °C.

In order to obtain information about the sensing ability of the manufactured sensors, the resistance of the samples was investigated from 100 to 500 °C. For dc resistance measurement of samples and to control the resistance of self-heater a test setup as depicted in Figure 1 was used. 20% O_2_ in N_2_ with 3 vol % H_2_O served as base atmosphere. NO (500 ppm), CO (500 ppm), H_2_ (1000 ppm) or NO_2_ (500 ppm) were added (see Figure 6). The temperature of the gas pipes was kept at 120 °C with an electrical heating hose. The total gas flow was adjusted to 1 L/min. The dc resistance of samples was recorded every 3 s by digital multimeter. The response to each component, *S*_component_, was calculated according to Equation (2). Here, *R*_g_ and *R*_a_ refer to the sample resistance before and after gas exposure, respectively. The best operation temperature for each gas component was found out that way.

(2) $$S_{component}=\frac{\left|R_{a}-R_{g}\right|}{R_{g}}$$

The response of the AD-coated SnO_2_ sensor to the above-mentioned gas components is shown in Figure 6. A typical volcano-shaped relation for all test gases with maximum between 300 °C and 400 °C was found in good agreement with results of Baik et al. [37]. While the sensor response to H_2_, *S*_H2_, is around 42 at 400 °C, *S*_NO_, *S*_CO_ and *S*_NO2_ are 3.3, 28, and 4.6, respectively. The response time (*t*_90_–*t*_10_) of the sensor to CO is around 11 s and to H_2_ it is lower than 2 s at 400 °C.

**Figure 7:**
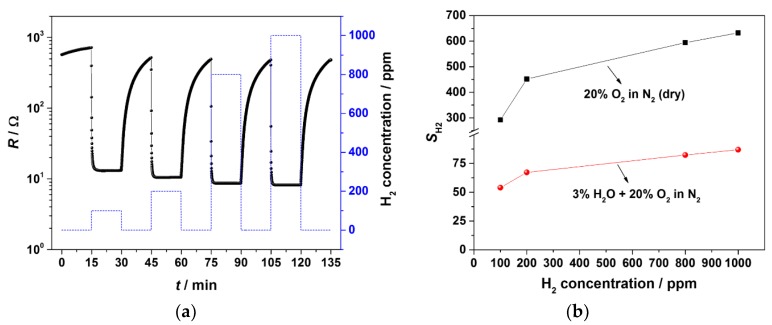
(**a**) Resistance of AD-coated SnO_2_ sensor under different H_2_ concentrations (100 to 1000 ppm) in base gas (3% H_2_O and 20% O_2_ in N_2_) at 400 °C (dotted line shows the concentration of H_2_), (**b**) comparison of the sensor response towards H_2_ in dry base gas and with 3% H_2_O added base gas.

Since the SnO_2_ sensor shows a high response and a short response time at 400 °C, the response of sensor to H_2_ from 100 to 1000 ppm was investigated at this operating temperature. In order to investigate the effect of water vapor on H_2_ sensitivity, the sensor was tested with 3% H_2_O and without water vapor. The result is depicted in Figure 7a,b. Figure 7a shows a resistance change of the sensor as a function of the H_2_ concentration in the presence of base gas (3% H_2_O and 20% O_2_ in N_2_). Although the recovery time of the sensor is quite long, the response is fast and reproducible even for 100 ppm H_2_. The comparison of H_2_ sensitivity of sensor with 3% H_2_O and without water is given in Figure 7b. It is clear from these results that the sensitivity towards H_2_ decreases in the presence of water vapor. The response of the sensor for 100 ppm H_2_ is 54, while for 1000 ppm H_2_ it is 87. It is observed that in the presence of water vapor in base gas, the conductance of SnO_2_ increased. Yamazoe et al. suggested that adsorption of water vapor always increases the conductivity of SnO_2_ and investigated the mechanism temperature-programmed desorption technique [38]. Barsan and Weimar suggested that molecular water and adsorption of hydroxyl groups might occur in the presence of water vapor between 100 and 500 °C [23]. A detailed explanation of the mechanism of the interaction of water vapor with the SnO_2_ surface can be found in reference [23].

The performance of the SnO_2_ sensor in general is related to crystal size, grain boundaries and compositional characteristics [26]. Yamazoe et al. correlated the effect of crystallite size of SnO_2_ with the sensitivity [39]. For crystal sizes of SnO_2_ being smaller than 6 nm, the response was found to increase strongly [39]. Sakai et al. fabricated SnO_2_ thin films (crystal size around 6 nm) of 100 to 300 nm thickness from a hydrothermally treated sol suspension and compared their response to 800 ppm CO and H_2_ [40]. They found that the sensor response to H_2_ is highly dependent on the film thickness while the response to CO is almost independent. The sensor response to H_2_ decreases when the film thickness is increased. Since the here-described sensor has a film thickness of about 1 µm and 35 nm crystal size, the lower response (*S*_H2_ = 56) to 800 ppm H_2_ is reasonable.

## 4. Conclusions

The AD process has the unique feature of room temperature processing of ceramic thick films. Since the powder properties such as particle size and morphology are decisive parameters for deposition success and film quality, we used a thermal pre-treatment to deposit nano-sized powders of tin (IV) oxide that lead untreated to a poor film quality. After the powder treatment well-adhering, dense AD films occurred. Coarsening of particles and crystallite size to a minimum required for deposition was done by a temperature step while too large particles that would harm the film were crushed by a subsequent milling step. As a result, we achieved homogeneous and translucent SnO_2_ thick-films as sensing layers on interdigital transducers. In several experiments, the sensing of various reducing gases was verified to be in good agreement with the literature. This study can serve as an exemplary attempt to make AD accessible to a wider variety of powders.

## Figures and Tables

**Table 1:** Deposition parameters for SnO_2_ films.

Carrier Gas	Mixtures of O_2_ and He
Gas consumption	2–5 L/min
Pressure in deposition chamber	<0.1 mbar
Pressure in aerosol chamber	55–160 mbar
Size of nozzle orifice	10 mm × 0.5 mm
Stand-off distance of nozzle to substrate	2–5 mm
Scanning speed	2–5 mm/s
